# The Sordariomycetes: an expanding resource with Big Data for mining in evolutionary genomics and transcriptomics

**DOI:** 10.3389/ffunb.2023.1214537

**Published:** 2023-06-30

**Authors:** Zheng Wang, Wonyong Kim, Yen-Wen Wang, Elizabeta Yakubovich, Caihong Dong, Frances Trail, Jeffrey P. Townsend, Oded Yarden

**Affiliations:** ^1^Department of Biostatistics, Yale School of Public Health, New Haven, CT, United States; ^2^Korean Lichen Research Institute, Sunchon National University, Suncheon, Republic of Korea; ^3^Department of Plant Pathology and Microbiology, The Robert H. Smith Faculty of Agriculture, Food and Environment, The Hebrew University of Jerusalem, Rehovot, Israel; ^4^Institute of Microbiology, Chinese Academy of Sciences, Beijing, China; ^5^Department of Plant Biology, Michigan State University, East Lansing, MI, United States; ^6^Department of Plant, Soil and Microbial Sciences, Michigan State University, East Lansing, MI, United States; ^7^Department of Ecology and Evolutionary Biology, Program in Microbiology, and Program in Computational Biology and Bioinformatics, Yale University, New Haven, CT, United States

**Keywords:** Sordariomycetes, evolution, genomics, transcriptomics, Big Data, *Neurospora*, *Fusarium*, *Trichoderma*

## Abstract

Advances in genomics and transcriptomics accompanying the rapid accumulation of omics data have provided new tools that have transformed and expanded the traditional concepts of model fungi. Evolutionary genomics and transcriptomics have flourished with the use of classical and newer fungal models that facilitate the study of diverse topics encompassing fungal biology and development. Technological advances have also created the opportunity to obtain and mine large datasets. One such continuously growing dataset is that of the Sordariomycetes, which exhibit a richness of species, ecological diversity, economic importance, and a profound research history on amenable models. Currently, 3,574 species of this class have been sequenced, comprising nearly one-third of the available ascomycete genomes. Among these genomes, multiple representatives of the model genera *Fusarium*, *Neurospora*, and *Trichoderma* are present. In this review, we examine recently published studies and data on the Sordariomycetes that have contributed novel insights to the field of fungal evolution *via* integrative analyses of the genetic, pathogenic, and other biological characteristics of the fungi. Some of these studies applied ancestral state analysis of gene expression among divergent lineages to infer regulatory network models, identify key genetic elements in fungal sexual development, and investigate the regulation of conidial germination and secondary metabolism. Such multispecies investigations address challenges in the study of fungal evolutionary genomics derived from studies that are often based on limited model genomes and that primarily focus on the aspects of biology driven by knowledge drawn from a few model species. Rapidly accumulating information and expanding capabilities for systems biological analysis of Big Data are setting the stage for the expansion of the concept of model systems from unitary taxonomic species/genera to inclusive clusters of well-studied models that can facilitate both the in-depth study of specific lineages and also investigation of trait diversity across lineages. The Sordariomycetes class, in particular, offers abundant omics data and a large and active global research community. As such, the Sordariomycetes can form a core omics clade, providing a blueprint for the expansion of our knowledge of evolution at the genomic scale in the exciting era of Big Data and artificial intelligence, and serving as a reference for the future analysis of different taxonomic levels within the fungal kingdom.

## Traditional models have served as stepping stones in evolutionary genomics

A model system—sometimes also referred to as a model species—is an optimized living non-human platform that can be easily accessed and manipulated (Davis, 2003b; Yarden et al., 2003; Ankeny and Leonelli, 2020). The progress made in experimental and computational biology, which has contributed to strengthening the links between descriptive research and mechanistic understanding—especially by utilizing molecular biology and genetics—has been largely based on the employment of model systems. Traditional fungal models, including unicellular fungi such as *Saccharomyces cerevisiae* (Saccharomycetaceae, Saccharomycetales) and *Schizosaccharomyces pombe* (Schizosaccharomycetaceae, Schizosaccharomycetales), as well as filamentous fungi such as *Neurospora* (Sordariaceae, Sordariales) and *Aspergillus* spp. (Trichocomaceae, Eurotiales), have well-characterized morphology, have been intensively studied genetically, and, in some cases, are also of economic importance (Yarden, 2007; Yarden, 2016). One of the primary bases for the selection of some of these organisms has been their amenability to developmental, physiological, and genetic manipulations. Currently, technological capabilities have progressed to the point where additional species can be studied and manipulated with increasing ease, providing the option to address specific questions and problems in a growing number of organisms (Goldstein and King, 2016).

The classic model systems, including yeasts, *Neurospora*, and *Aspergillus*, were among the first set of targets when genome sequencing became technically and economically feasible two decades ago, and omics data from these early models profoundly reshaped modern fungus research (Feldmann, 1999; Galagan et al., 2005; Sunnerhagen and Piskur, 2006; Dunlap et al., 2007; Jones, 2007; Nowrousian, 2014; Ellena and Steiger, 2022). Although the roles of classic fungal models in general biology have become increasingly confined (Davis, 2003a), new insights from the genomics of fungal models continue to attract interest in basic fungal biology, especially as they relate to evolutionary biology and ecology, including issues of diversity, invasive species, the impact of climate change, speciation and species concept, circadian clocks, and pathogenicity (Dyer and O’Gorman, 2012; Stukenbrock, 2013; Plissonneau et al., 2017; Stajich, 2017; Boekhout et al., 2021; North et al., 2021; Peris et al., 2022; Feurtey et al., 2023; Kelliher et al., 2023). In addition, these well-studied models have served as references for the annotation of less-studied and rarer systems, including many non-model species (Russell et al., 2017; Tworzydlo and Bilinski, 2019), which range from taxonomically related species to ecologically and economically relevant species. However, the rapid development of comparative genomics techniques (**Box 1**) has facilitated the analysis of new fungal genomes as they have been sequenced (Sivashankari and Shanmughavel, 2007) and has also greatly impacted the analysis of genomes from under-represented fungal classes, especially the basal lineages. These advances in knowledge have improved our understanding of the origin and diversity of the fungal kingdom (Stukenbrock and Croll, 2014; Wang et al., 2016d; Dornburg et al., 2017; Stajich, 2017; Chang et al., 2021). Here, we review recent advances in comparative omics in the class Sordariomycetes, focusing on how model species in this class have contributed to evolutionary biology and ecology, and how this class can be developed as a template for obtaining novel and extended insights concerning the fungal kingdom.

## Clusters of well-studied models with Big Data to bridge large gaps in evolutionary genomics

One of the main drivers of progress in large-scale comparative genomics research on fungi has been the fungal research core at the Joint Genome Institute, where the 1000 Fungal Genomes Project has been pursued (Grigoriev et al., 2011; Grigoriev et al., 2014). Investigation of genomics and available transcriptomic data for a highly inclusive sample of fungal genomes has revealed that evolutionary convergence may occur across large phylogenetic distances (Merényi et al., 2020). Comparative genomics in mushroom-forming fungi has produced insights into the evolution of mycorrhizal symbiosis, wood-decay mechanisms, and morphological development (Riley et al., 2014; Nagy et al., 2016; Miyauchi et al., 2020; Sánchez-García et al., 2020; Virágh et al., 2022). For example/instance, using comparative genomics to examine a more focused group within the Ustilaginaceae, researchers have reported that the gain and loss of effector genes, including orphan and lineage-specific selected genes, are probably the most important determinants of the host specificity of smut fungi (Benevenuto et al., 2018).

Widespread advances in omics technologies, such as genomics, transcriptomics (whole-genome RNA expression profiling), proteomics (genome-wide study of proteomes), and even metabolomics, have further enabled the study of evolutionary genomics at an extraordinarily detailed molecular level. A key advantage of these technologies is their ability to provide a more granular understanding of evolution within gene families and functional groups, and to elucidate the roles that these families and groups play in large molecular processes. An example of an omics-based integrative approach is the demonstration of the presence of highly conserved class-dependent sugar metabolism pathways (Li et al., 2022). Such analyses, in addition to substantiating the taxonomic placements of the species involved, can also assist in providing guidelines concerning the challenges and limitations of transferring metabolic pathways between species for industrial applications. In the course of analysis of Bayesian networks derived from transcriptomics data on *Neurospora* and *Fusarium* (Nectriaceae, Hypocreales), dispensable chromosomes related to pathogen specificity, very large effector classes, and the dynamic regulation of meiotic silencing and transcription factor networks during sexual reproduction were identified in these models (Wang et al., 2014; Trail et al., 2017; Wang et al., 2018a). A proteogenomics study on the model *Sordaria macrospora* (Sordariaceae, Sordariales) identified new genes and previously unknown posttranscriptional modifications (Blank-Landeshammer et al., 2019). As omics data from different species is increasingly accumulated, the evolutionary integration of multiple models will be essential in analyzing genomic divergences between distinct fungal clades. Computational approaches to the processing of large-scale omics data and data integration methods that focus mainly on a single model are continuously being developed (Winkler, 2020; Duruflé and Déjean, 2023; Shave et al., 2023; Vasaikar et al., 2023). In addition, systems biological approaches—especially those that refine networks *via* statistical modeling for large datasets with a small cohort size—have proved to be useful in the interpretation of omics data (Culibrk et al., 2016; Karahalil, 2016; Maghuly et al., 2022). These techniques can improve and ease the analysis of “unmatched” multimodal data and provide enhanced overall performance compared with traditional methods that typically do not enable the visual and quantitative or semiquantitative interpretation of gene–gene interactions.

To the apt phrase “Nothing in biology makes sense except in the light of evolution” (Dobzhansky, 1973) can be added “Nothing in evolution makes sense except in the light of genomics” (Shapiro, 2016). Evolutionary biologists have benefited from advances in diverse aspects of the sciences, and omics methods have become the latest powerful toolbox that can be used to clarify the workings of novel features that have hitherto been inexplicable until the last few decades (Shapiro, 2016). Proceeding from genic phylogenies to phylogenomics, the evolutionary histories of major known lineages in fungi have been successfully and robustly resolved, enabling the retrospective tracking of molecular events along the history of fungal evolution (Nowrousian, 2014; Heitman et al., 2017; Pöggeler and James, 2023). In another example, the *Neurospora* community has produced a systematic gene disruption strain collection (“the *Neurospora* knockout collection”), targeting nearly the entire genome, and made it available to the public. There are now over 11,000 gene-disrupted strains available at the Fungal Genetics Stock Center (Colot et al., 2006; Dunlap et al., 2007; Collopy et al., 2010). Meanwhile, advances in gene-manipulation techniques, especially the recently developed CRISPR-Cas9 gene editing system, have dramatically improved our ability to identify the causal mechanisms that link genes and phenotypes in diverse species. Many functional gene groups and metabolic pathways have also been subjected to systematic gene manipulations in various fungal species, and conserved metabolic pathways and key elements in development regulatory circuits have been identified in individual fungal species, many of which are model species. Thus, the progress made in the areas of fungal phylogeny, genomics, and functional genetics has provided a strong foundation for the use of a broader, multispecies model to address the mechanistic bases of complex traits in a diverse range of organisms within particular environmental, developmental, social, and/or genomic contexts.

## Sordariomycetes is a large class exhibiting broad diversity, accumulated over time

Advances in genome sequencing and omics data analysis have facilitated further comparative studies, which have offered new insights into fungal evolution (Nagy et al., 2016; Merényi et al., 2020; Miyauchi et al., 2020; Sagita et al., 2021; Sierra-Patev et al., 2023). Examples covering a wide range of significant discoveries made through comparative genomics include to resolve evolutionary histories among early fungal lineages (James et al., 2020), to propose an important molecular mechanism associated with flagellated zoospores in the chytrid fungus *Blastocladiella emersonii* (Blastocladiaceae, Blastocladiales; Galindo et al., 2022), to identify horizontal transfer of a large and toxic secondary metabolic gene cluster between *Podospora* and *Aspergillus* (Slot and Rokas, 2011), to reveal mobile pathogenicity chromosomes in *Fusarium* (Ma et al., 2010), and to discover a small genome of a species within the mycoparasitic genus *Escovopsis* (Hypocreaceae, Hypocreales) that rely on their hosts for some key cellular functions (de Man et al., 2016). This progress has prompted efforts to identify missing links, e.g., non-model species, which can be sequenced and manipulated to address specifically defined biological and evolutionary questions (Russell et al., 2017). In addition to providing annotation references for non-model species, a set of model species forms the phylogenetic backbone for a better understanding of the ways in which species have evolved at the genomic level. Comparative genomic analysis, aided by the inclusion of well-studied models, identifies divergence in genome content and structure, gene copy number and sequence, gene synteny, and evolutionary history, and also identifies mobile elements and non-coding regions in non-model genomes (Sunnerhagen and Piskur, 2006; Sivashankari and Shanmughavel, 2007).

**Figure 1:**
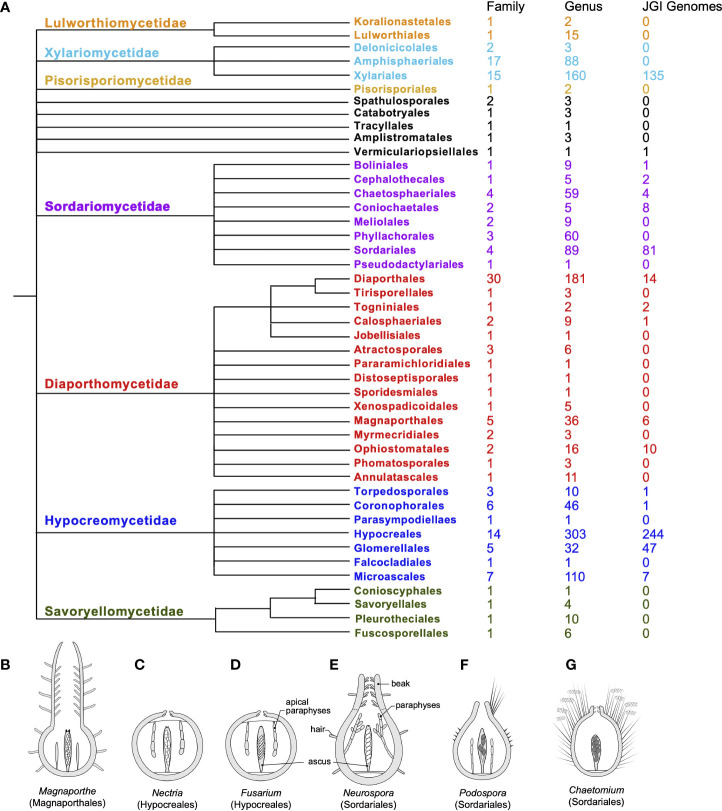
Systematics, genome availability, and some model species for the Sordariomycetes. **(A)** Updated systematics of the class Sordariomycetes, composed of seven subclasses (color-coded, alongside the current numbers of family, genus, and genome sequences available in the JGI fungal genome database for each order). The ordinal phylogeny was adapted from a phyloT tree of NCBI taxonomy in the Sordariomycetes and modified in accordance with the latest arrangement by Hyde et al. (2020). **(B–G)** Comparative morphology of fruiting bodies (perithecia) of model species in six genera: **(B)** *Magnaporthe*, **(C)** *Nectria*, **(D)** *Fusarium*, **(E)** *Neurospora*, **(F)** *Podospora*, and **(G)** *Chaetomium*, including a longitudinal section through the outer fruiting body, a single representative mature ascus with ascospores, and representative paraphyses (sterile hyphae associated with asci). Each perithecium harbors tens or hundreds of asci. All genera, except *Magnaporthe* and *Chaetomium*, forcibly discharge their spores. *P. anserina* produces only four spores per ascus; all others generally produce eight (Trail, 2013; after Trail and Seminara, 2014).

Fungi in the class Sordariomycetes exhibit high levels of ecological diversity. They include saprotrophs on decaying substrates as well as endophytes and parasites on plants, insects, vertebrates, and even fungi. They are also capable of producing a diverse array of secondary metabolites under different conditions (Charria-Girón et al., 2022). The profiles of the secondary metabolites produced can be closely correlated with their phylogeny. However, genomic and phylogenomic analyses strongly suggest that even the genomes of well-studied species harbor secondary metabolite biosynthesis gene clusters, the structures and functions of which have yet to be determined (Atanasov et al., 2021). It has been suggested that the ancestral status of the ecology for Sordariomycetes is saprotrophic or parasitic (Zhang et al., 2006; Zhang and Wang, 2015). In fact, Sordariomycetes is one of the largest classes within the Ascomycetes, and, along with progress in molecular phylogeny, the systematics of this class has been revised multiple times, reflecting the complexity of evolutionary histories within the class. In the most recent comprehensive review of the class (**Figure 1A**), a total of seven subclasses, 45 orders, 167 families, and 1,499 genera (with 308 genera *incertae sedis*) are recognized within Sordariomycetes, representing dramatic diversity in ecology and developmental biology (Maharachchikumbura et al., 2015; Maharachchikumbura et al., 2016; Hyde et al., 2020). Among the Sordariomycetes, there are several species that serve as classic models of filamentous fungi in the genera, such as *Neurospora* and *Podospora* (Podosporaceae, Sordariales); *Sordaria* species for developmental genetics; *Fusarium*, *Magnaporthe* (Magnaporthaceae, Magnaporthales), and the *Cordyceps* (Cordycipitaceae, Hypocreales) species for pathogenesis; and *Chaetomium* (Chaetomiaceae, Sordariales) and *Trichoderma* (Hypocreaceae, Hypocreales) species for metabolics and fermentation.

## The need for the evolutionary integration of multiple models

### Classic genetic models

Among the several Sordariomycetes fungi that have been utilized as models, *N. crassa* is perhaps the most widely examined, and was the first multicellular fungus to have its genome sequenced and annotated (Borkovich et al., 2004; Dunlap et al., 2007). It is a non-pathogenic saprotroph that grows vegetatively and can be cultured in most defined and natural media, and, as such, remains a widely used model that facilitates research across a worldwide community, which has addressed a broad range of questions in general and fungal biology (Perkins, 1992). The species has a distinguished history, including its role in the formulation of the “one gene—one enzyme” hypothesis proposed by Beadle and Tatum (1941).

Closely related to *N. crassa*, *N. tetrasperma* is pseudohomothallic and employs a mating strategy that combines selfing with occasional outbreeding (Merino et al., 1996; Sun et al., 2017). *N. tetrasperma* provides a model for study of both the genomic and the evolutionary consequences of asexuality and inbreeding (Sun et al., 2012; Samils et al., 2013; Idnurm et al., 2015). These consequences cannot be studied in obligate outbreeders alone, such as *N. crassa* and other popular genetic models. A pangenomic analysis of 92 genomes from eight phylogenetically and reproductively isolated lineages of *N. tetrasperma* has provided experimental evidence for the role of introgression as a mechanism for the maintenance of mating-type-determining chromosomal regions (Corcoran et al., 2016).

Interestingly, *Neurospora* species are also known as postfire fungi, as they are often spotted in the forest after a fire, a unique environment shared with certain other pyrophilous fungi (Hansen and Pfister, 2006; Wicklow, 2018), such as *Geopyxis carbonaria* (Pyronemataceae, Pezizales) and some species of *Pyronema* and *Peziza* (Pezizales). Another easily maintained and genetically manipulated species, *Podospora anserina*, has long been used as a model to study aging, meiosis, sexual reproduction, and heterokaryon formation in fungi (Philipp et al., 2013; Silar et al., 2019; Hartmann et al., 2021a; Lelandais et al., 2022). *P. anserina* and *N. crassa* genomes share over 60%–70% similarity in orthologous proteins, making them a powerful pair of models that can be used as references for comparative genomics (Espagne et al., 2008; Paoletti and Saupe, 2008).

Yet another intensively studied developmental genetic model in the Sordariomycetes is *S. macrospora*, with over 100 available developmental mutants (Nowrousian et al., 2010; Nowrousian et al., 2012; Blank-Landeshammer et al., 2019; Teichert et al., 2020). Similar to species of *Neurospora* and *Podospora*, *S. macrospora* grows quickly under laboratory conditions. The fact that *S. macrospora* is homothallic can be advantageous for sexual and developmental synchronization and for maintenance of a homogeneous genetic background.

### Models for fungal pathogenesis

A small number of Sordariomycetes fungi, including *Fusarium* and *Magnaporthe* species, are models for fungal pathogenesis; over the past decades, *Fusarium* research has advanced our understanding of these fungi. *Fusarium* is a large genus, including some anamorphic fungi, for which sexual development and reproduction has never been observed (O’Donnell et al., 2015). This genus harbors important plant pathogens, producers of a wide range of fungal chemicals, and causal agents of opportunistic mycoses in humans (Suga and Hyakumachi, 2004). *F. graminearum sensu lato* is a species complex whose members cause devastating diseases in small grains and mycotoxin contamination worldwide (Wang et al., 2010; Wang et al., 2011; Garmendia et al., 2018; Chai et al., 2022; Del Ponte et al., 2022). *F. graminearum* sensu stricto refers to the major disease-causing species in the USA (and a few other places); as this was the subject of one of the first genomics projects (Cuomo et al., 2007), it has since been frequently used in many comparative studies (Khan et al., 2020; Rampersad, 2020; Teli et al., 2020). This fungus has been intensively studied as a cosmopolitan model for fungal pathogenesis of crop plants. The largest source of inoculum for disease outbreaks originates from the primary inoculum, airborne sexual spores (forcibly discharged from fruiting bodies), with aboveground asexual spores (mainly splash-dispersed) providing a local secondary inoculum (Ingold and Dring, 1957). For this reason, the species *F. graminearum* has become a model for the study of perithecium development on host plants (Kim et al., 2022) and the mechanism of forcible ascospore discharge (Trail et al., 2005; Trail, 2007; Cavinder et al., 2012; Trail and Seminara, 2014; David et al., 2016). Recently, *F. graminearum* has become the first plant-pathogenic fungus documented to produce biofilms in association with plant colonization (Shay et al., 2022). Although fungicide applications are the main source of control over the disease, there are no strongly resistant varieties of wheat or barley available. In addition to being infamous crop pathogens, *Fusarium* spp. are also considered to be severe threats to human health and are listed in the WHO fungal priority pathogens list to guide research, development, and public health action (World Health Organization, 2022; Rodrigues and Nosanchuk, 2023). As in the case of *Neurospora* spp., comparative genomic analysis of *Fusarium* spp. has also been instrumental to the field’s understanding of the evolution of sexual development (Trail et al., 2017). One example is the identification and analysis of horizontally transferred lineage-specific genomic regions related to pathogenicity (Ma et al., 2010).

*Magnaporthe* species cause devastating crop diseases. *M*. *grisea*, the causal agent of rice, was sequenced in 2005 (Dean et al., 2005). Rice blast causes significant economic loss: worldwide, enough rice to feed tens of millions of people is destroyed by rice blast every year (Kumar et al., 2020). Closely related to *M. grisea*, *M. oryzae* attacks wheat, causing wheat blast, an emergent fungal disease that probably evolved through a series of “host jumps” and that possesses core chromosomes and mini-chromosomes with distinct evolutionary histories (Hossain, 2022). A genomic surveillance study of over 500 strains from diverse geographic regions and host types has revealed the adaptation and development of fungicide resistance in a pandemic clonal lineage of *M. oryzae* (Latorre et al., 2023; Rhodes, 2023). Infections occur when fungal spores land on and invade wheat leaves using an appressorium—a specialized infection cell formed during spore germination. The process of the development of appressoria has recently been investigated intensively using genome-wide analysis (Lv et al., 2022; Wang et al., 2022a; Fan et al., 2023; Miguel-Rojas et al., 2023; Qian et al., 2023; Rogers and Egan, 2023). Diverse ecologies have been reported for species of the Magnaporthales, ranging from saprotrophs to cereal pathogens to likely root endophytes (Luo et al., 2015). The Sordariomycetes also include diverse entomopathogenic fungi, including the “zombie” fungus genus *Cordyceps* and the species of *Beauveria* (Cordycipitaceae, Hypocreales) and *Metarhizium* (Cordycipitaceae, Hypocreales), commonly used as biopesticides of pathogenic insects and arthropods. Omics-based investigation of these fungi is expanding rapidly (Zheng et al., 2011; Xiao et al., 2012; Pattemore et al., 2014; Valero-Jiménez et al., 2016; Wang et al., 2016a; Chen et al., 2019; Kato et al., 2022; Thananusak et al., 2022; Liu and Dong, 2023). Interestingly, some fungi, such as *Metarhizium* species, are capable of attacking hosts in both the plant and the animal kingdoms.

In addition to plant, arthropod, and human hosts, the Sordariomycetes also include a unique pathogenic group, mycoparasites, capable of parasitizing other fungi, including both pathogenic and economically beneficial species. These include species such as *Clonostachys rosea* (Hypocreales; Bionectriaceae), *Calcarisporium cordycipiticola* (Hypocreales; Calcarisporiaceae), *Lecanicillium fungicola* (Hypocreales; Cordycipitaceae), *Syspastospora parasitica* (Hypocreales; Hypocreaceae), and *Escovopsis weberi* (Hypocreales; Hypocreaceae) (Posada et al., 2004; Berendsen et al., 2010; de Man et al., 2016; Sun et al., 2020; Liu et al., 2021). Over the years, significant attention has been given to the study of mycoparasites belonging to the genus *Trichoderma*, which contains many species of significant value to agriculture and industry, and the genomics of which have been comprehensively examined (Schalamun and Schmoll, 2022). *Trichoderma reesei* is a unique species among other industrial fungi (Seidl et al., 2009; Druzhinina et al., 2011)*. T. reesei* (teleomorphic; *Hypocrea jecorina*) is a known industrial cellulolytic enzyme producer, making it a model system for the study of the regulatory mechanisms of plant cell wall-degrading enzymes; this work contributes to the biofuel and agricultural waste industries. *T. harzianum*, *T. asperellum*, and *T. asperelloides* are among the most thoroughly analyzed *Trichoderma* spp. and have been incorporated in biocontrol strategies (Druzhinina et al., 2011). These biocontrol species and other *Trichoderma* strains have been demonstrated to successfully control plant diseases by stimulating plant growth and development, increasing plant resistance to biotic and abiotic stresses, and directly parasitizing phytopathogenic fungi (Zeilinger and Omann, 2007; Atanasova, 2014; Guzmán-Guzmán et al., 2019; Poveda, 2021; Di Lelio et al., 2023; Woo et al., 2023). Moreover, *T. reesei* and *T. asperellum* are considered to be models for the industrial production of various secondary metabolites (Mukherjee et al., 2013; Schalamun and Schmoll, 2022) and biofungicides due to their mycoparasitic properties, which benefit plants challenged by other fungal pathogens (Kubicek et al., 2011).

The first full *Trichoderma* genome analyzed was that of *T. reesei* (Martinez et al., 2008)*. T. asperellum* and *T*. *asperelloides* (along with others) were analyzed later (Berka, 2017; Druzhinina et al., 2018; Gortikov et al., 2022a), well after the genomes of *Neurospora* and *Fusarium* species (Druzhinina et al., 2018). The recently sequenced genome of *T. asperelloides* contributed to the determination of its correct taxonomic identity, in contrast to its previous identification as the closely related *T. asperellum*, a model for biological control (Podder and Ghosh, 2019; Gortikov et al., 2022a). Currently, there are more than 90 *Trichoderma* genomes that have been sequenced and released, making it among the most sequenced genera in the class; this is an achievement that can be attributed to a combination of industrial and academic efforts. Comparative genomic insight into *Trichoderma* spp. is fairly recent. However, insights into the core genome of this genus, potential heterothallic reproduction, and the abundance of species-specific orphan genes (Kubicek et al., 2019) have provided a strong foundation for further class-based comparative genomics with other Sordariomycetes. One species of Sordariomycetes, *Calcarisporium cordycipiticola* (Calcarisporiaceae, Hypocreales), is a mycoparasite of *Cordyceps militaris*; mycoparasitism causes devastating diseases of fruiting cultivation. Phylogenomic analysis has highlighted the fact that *C. cordycipiticola* was evolutionarily close to its host *C. militaris*, and that they diverged after a split with the *Trichoderm*a genus. Comparative genomic and transcriptome analyses have provided insights into the origin of the pathogen and the mycoparasitic interactions of two species from sister families (Liu et al., 2021; Liu and Dong, 2023).

### Promising new models for fungal biology

Dramatic divergences in the development and biology of Sordariomycetes can be observed even among closely related species; thus, the class offers excellent systems for assessment of the molecular genetic basis of the evolution of these species. Among these promising systems, *Chaetomium* spp. fungi exhibit a frequently homothallic lifestyle, a general lack of asexual reproduction of conidia, enriched secondary metabolism synthesis, and an ability to thrive in highly humid environments (Wang et al., 2016b; Zámocký et al., 2016; Wang et al., 2019a). All of these traits are highly divergent in the genus, rendering these species especially informative regarding rapid evolution in a comparative evolution context. The *Chaetomium* spp. are cosmopolitan saprotrophs and endophytes that are capable of dominating diverse ecological niches, including some extreme environments, evoking interest in their genomic adaptation for survival and dispersal. Species of *Chaetomium* are common contaminants in indoor environments and are considered to be health hazards (Andersen et al., 2011; Miller and McMullin, 2014), causing symptoms of rhinitis and asthma when they infect humans (Mackenzie, 1979; Vesper et al., 2007; Hassett et al., 2009; Green et al., 2014). *C. globosum* is a model for the industrial production of secondary metabolites, and is also generally considered to be a species complex (Asgari and Zare, 2011; Wang et al., 2016b). Chaetomium species probably shared thermophilic ancestors, consistent with the thermophilic basal nature of its most diverged lineage (Zámocký et al., 2016). The genome sequence of *C. globosum* was made publicly available in 2015 (Cuomo et al., 2015). Since then, the taxonomy and phylogenetics of the genus have been undergoing reassessment and revision (Wang et al., 2022c).

These models represent some of the diverse genera within the Sordariomycetes that have been studied intensively. The availability of many genome sequences that are closely related to established and emerging model species represents a substantial contribution to analyses of the ecological traits of these organisms and their close relatives, which can in turn provide substantial insight. Currently, there are 570 Sordariomycetes genomes (covering 136 genera or high ranks, with more than 330 identified species and 75 unidentified species) in the JGI fungal genome database (mycocosm.jgi.doe.gov/), in addition to the comparative tools available *via* that portal (**Figure 1A**). Among these genomes, 189 cover more than 60 species in four genera, including 81 *Fusarium* and 94 *Trichoderma* species. Many additional Sordariomycetes genomes are available *via* the NCBI (National Center for Biotechnology Information), with 3,574 genomes (nearly one-third of the total of 11,083) being published and drafted ascomycetes genomes; these include 1,359 *Fusarium*, 117 *Neurospora*, 110 *Trichoderma*, and 14 *Chaetomium* genomes. Many of these genomes are for species that are culturable, produce simple morphologies, and are often either pathogenic or economically important. The ecological and industrial relevance of pathogenic members of Sordariomycetes and those producing valuable fungal products has been a major driver of the extensive sequencing of many non-model genomes in this fungal class. Some of these non-model species are emerging as “new” models and, unlike the classic models that have undergone intensive investigation in biology and genetics, established during the pre-genomics era, these species are often studied under a reverse approach, with investigation originating in genomics and then extending to genetics and the analysis of biological traits. For example, the genomes of a large number of species of Xylariale have recently been sequenced, and comparative genomics has revealed genetic changes that are probably associated with bioactivities, diverse lifestyles, environmental adaptations, and selective pressure in these “mystical”-looking fungi (Robinson et al., 2020; Wibberg et al., 2020; Franco et al., 2022; Fricke et al., 2023). At the same time, the annotation of classic model genomes, such as those of *Neurospora*, *Podospora*, and *Sordaria* genomes, has undergone continuous improvement, in part on the basis of information obtained from newly sequenced genomes of the same or closely related species (Teichert et al., 2014; Gladieux et al., 2015; Blank-Landeshammer et al., 2019; Teichert et al., 2020; Lelandais et al., 2022; Rodriguez et al., 2022; Vittorelli et al., 2023). These accumulating data continue to contribute to a new era of evolutionary genomics and transcriptomics at further taxonomic levels within the Sordariomycetes class.

## New details and new omics data on the Sordariomycetes continue to accumulate in an accelerating manner

Representatives of the major fungal lineages on the tree of life have now had their genomes sequenced, and the better-annotated genomes of model species have been used as references. Thus, model species can function as genomic information hubs to bridge the gaps between these well-studied organisms and the relatively novel characteristics of non-model species (Wang et al., 2018a; Teichert et al., 2020). Associations between the various different ecological and developmental models are of special interest, and within Sordariomycetes, it has been suggested that there is a strong correlation between the evolution of senescence and ephemeral substrate usage (Geydan et al., 2012). Sexual reproduction in the Sordariomycetes involves the formation of perithecium, a round-to-flask-shaped structure with a pore through which meiotic spores are discharged. The morphological details of the perithecium can differ dramatically among species within the class (**Figures 1B–G**). Sexual development has been examined in *Neurospora*, *Podospora*, and *Sordaria via* genomics- and transcriptomics-based analyses for over a decade (Bidard et al., 2011; Ellison, 2011; Dirschnabel et al., 2014; Teichert et al., 2014; Idnurm et al., 2015; Corcoran et al., 2016; Xie et al., 2017; Grognet et al., 2019; Shen et al., 2019; Hartmann et al., 2021b), with emphasis on the functions and evolution of mating loci, secondary metabolism, and stress responses associated with sexual reproduction.

### Evolutionary developmental biology

Divergence in gene expression has long been considered to play a critical role in developmental adaptations during organismal evolution (King and Wilson, 1975; Carroll, 2005; Hodgins-Davis et al., 2015; Diaz et al., 2023). The incorporation of divergence in sexual morphology within comparatively closely related taxa could improve the precision and accuracy of reconstructions of ancestral gene expression, yielding expansions to the number of associations between shared morphologies and shared transcriptional profiles. Comparative genomics and evolutionary transcriptomics focusing on Sordariomycetes models has revealed the genes and regulatory networks that are critical for sexual development in these fungi, including non-coding sequences and genes that were previously uncharacterized (Trail et al., 2017; Kim et al., 2018; Kim et al., 2019; Lütkenhaus et al., 2019). In addition, novel genes that affect perithecial development have been identified in *M. oryzae* and *N. crassa*, which have diverged functionally and transcriptionally from their orthologous counterparts in *F. graminearum* (Kim et al., 2022). These novel genes are predicted to be “young” (i.e., present only in a recently diverged clade of species) and tend to be involved in lineage- or species-specific functions. Phenotypic study guided by comparative genomics among three distantly related filamentous fungi has identified two genes (predicted to be chromatin modifiers) that play roles in the sexual development of *S. macrospora* (Lütkenhaus et al., 2019).

### Evolutionary ecology

**Figure 2:**
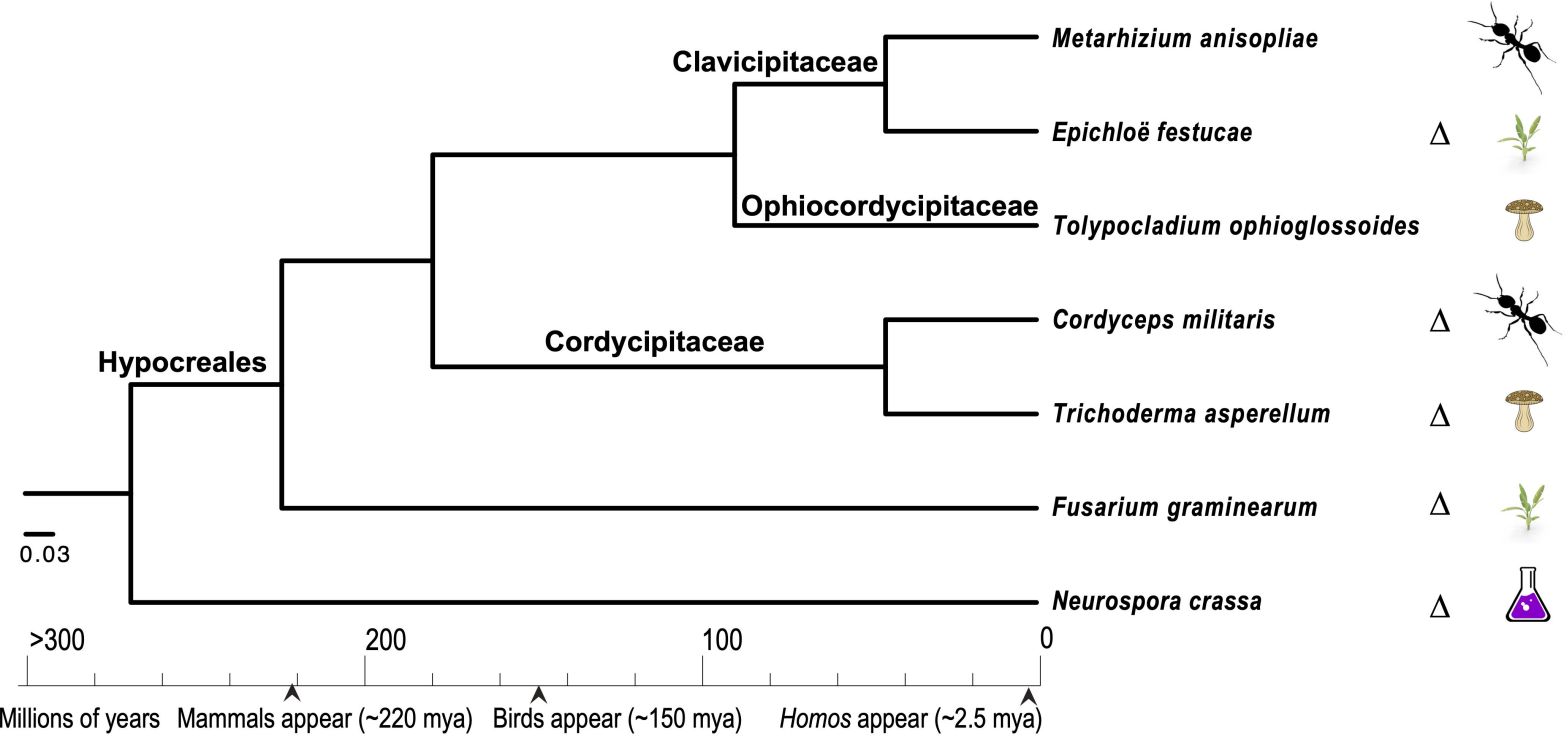
Phylogenetic relationships among seven closely related taxa in the Hypocreales, inferred with concatenated RPB1 and RPB2 protein sequences using a Bayesian approach (Ronquist et al., 2012) to investigate the repeated evolution of parasitism on specific host types. These species include fungi that attack insects (entomopathogenic species), plants (phytopathogenic species), other fungi (mycopathogenic species), and non-pathogenic species, including endophytic and experimental model species. The classification follows Zhang et al. (2006) and Geiser et al. (2006), and the calibration points used are those of Taylor and Berbee (2006). Species in which genetic manipulations have been well developed are marked with a delta symbol, “Δ”.

Comparative genomics approaches using these model species have also been pursued in order to understand the evolution of diverse ecologies among the Sordariomycetes, especially those that are related to pathogenesis (**Figure 2**). Among the pathogenic Sordariomycetes, species of *Fusarium* have been intensively studied for their diverse genome structures (Brown and Proctor, 2013; Ma et al., 2013; Zhang, 2019; Mir et al., 2023). Research on entomopathogenic Sordariomycetes, represented by *Cordyceps*, *Beauveria*, and *Metarhizium* species, has been greatly enhanced by the availability of genomes of these species (Gao et al., 2011; Pattemore et al., 2014; Valero-Jiménez et al., 2016; Kato et al., 2022; Liu and Dong, 2023; Sant Anna Iwanicki et al., 2023). *Neurospora* species have been examined as metabolic models of the biodegradation of cellulose and other plant organics (Feldman et al., 2019; Liu et al., 2020; Wu et al., 2020; Huberman et al., 2021), as well as providing some of the most tractable models for the circadian clock and fungal responses to environmental factors, such as light and temperature (Wang et al., 2016c; Dekhang et al., 2017; Wang et al., 2018b; Kelliher et al., 2020; Kelliher et al., 2023). Based on transcriptomics data from *N. crassa* grown on five different crop residues, researchers have discovered roles for a sporulation regulator *rca-1* in lignocellulose production (Wang et al., 2015). Additional roles in allorecognition have also been identified for *cwr-1*, a putative chitin polysaccharide monooxygenase, in *N. crassa* (Detomasi et al., 2022). Three studies have investigated the associations between secondary metabolite clusters (SMCs) and reproduction in two fungal models, *N. crassa* and *C. globosum*, and reported on the activities of 20 and 24 SMCs in these models, respectively—a manageable set for separate investigation of the roles of SMCs across the life cycle of these two models (Wang et al., 2019a; Wang et al., 2019b; Wang et al., 2022d). Bayesian regulatory networks have been reconstructed using transcriptomic data for *N. crassa* and *C. globosum*. These networks exhibit divergences in associations among genes associated with conidiation and heterokaryon incompatibility between *N. crassa* and *C. globosum*, supporting the theory that there is an evolutionary history of loss of conidiation in the latter, putatively due to unfavorable combinations of heterokaryon incompatibility in homothallic species. A recent study focused specifically on conidial germination in two model pathogens, *F. graminearum* and *M. oryzae*, on artificial medium and on hosts, as conidial germination represents the key stage in the initiation of fungal attack (Miguel-Rojas et al., 2023). The authors’ analyses revealed new and important aspects of early fungal ingress in *F. graminearum* that can form the basis for improvement of antifungal strategies, including a comparative gene expression finding that toxisomes are not fully functional until after penetration of the host cells. Based on a study using near-synchronous germinating cultures of *T. asperelloides* (a strain used in biocontrol that was formerly identified as *T. asperellum*), it has been reported that the transcript abundance of half of the annotated genome is reduced during early conidial germination (Grotikov et al., 2022b). In this study, it was also discovered that the expression of the chitin synthase and glucan elongase families is significantly increased during germination in the presence of a basidiomycete host, *Rhizoctonia solani* (Ceratobasidiaceae, Cantharellales), indicating that host recognition can occur during the early stages of mycoparasite development (Grotikov et al., 2022b).

### Genome evolution

Advances in fungal genomics have produced new insights regarding genome evolution in the kingdom. Mycovirus research has greatly benefited from the availability of a large number of fungal genomes and the development of comparative genomics (Kotta-Loizou, 2019; Myers et al., 2020). Mycoviruses have been the focus of biocontrol against fungal pathogens (Yu and Kim, 2020; Schalamun and Schmoll, 2022). More than 10 different families of viruses have been isolated from fungal hosts, including double-stranded RNA (dsRNA), positive-sense and negative-sense single-stranded RNA, and circular single-stranded DNA (ssDNA) viruses (Applen Clancey et al., 2020). The presence of viruses in *Fusarium* and *Trichoderma* spp. has been particularly well documented (Yun et al., 2016; Li et al., 2019a; You et al., 2019; Yu and Kim, 2020; Wang et al., 2022b). A recent study investigated the presence of viruses in multiple *Neurospora* species and identified various RNA viruses from *N. crassa* and other *Neurospora* spp. using transcriptomics data. The study further established *N. crassa* as a virus host model for use in the study of virus–host interactions and virology in fungi (Honda et al., 2020). The authors also demonstrated that *N. crassa* relies on transcriptional and posttranscriptional regulation to restrict virus replication for at least some of its genes.

Posttranscriptional regulation is another intriguing genome-wide editing mechanism, but how has such a widely distributed mechanism evolved and been maintained across the diverse fungal genome? One example of posttranscriptional regulation is A-to-I editing, which has recently been discovered to be associated with sexual development in filamentous fungi (Bian et al., 2019). Some of these A-to-I-editing filamentous fungi are Sordariomycetes, including *N. crassa* (Liu et al., 2017), *S. macrospora* (Teichert et al., 2017), and *F. graminearum* (Liu et al., 2016). In *M. oryzae*, *N*^6^-Methyladenosine (m^6^A) RNA methylation, the most common modification of RNA at the post-transcriptional level in eukaryotes, has been found to be important for various aspects of fungal biology, such as vegetative growth, conidiation, and pathogenicity (Shi et al., 2019). Although disruption of m^6^A factors does not affect sexual development in *M. oryzae* and *F. graminearum*, overexpression of the *m^6^A* writer (the ortholog of the gene coding for *IME4* in yeasts) causes delayed sexual development in *F. graminearum*, suggesting that m^6^A modification may alter developmental processes in filamentous fungi (W. Kim, unpublished). RNA modification, such as m^6^A, can be detected using direct RNA sequencing, whereby the modification sites can be profiled at nucleotide resolution (Leger et al., 2021). All these new discoveries using the Sordariomycetes models can be expected to evoke interest in exploring model fungal clades that can comprehensively illuminate these fundamental features.

### Climate change

The application of evolutionary approaches to genomic data from fungi may also help us to understand some impacts of climate change. Fungi are widely distributed and abundant in almost all ecosystems—even in extreme environments—and have been referred to as “climate warriors” in recognition of the important ecological roles that they play in the recycling of carbon, nitrogen, and other nutrients (Stajich, 2017; Averill et al., 2018; Tiquia-Arashiro and Grube, 2019; Pöggeler and James, 2023). Their presence and activity are considered to be useful indicators in relation to long-term climate change due to their sensitivity to surrounding conditions, such as changes in light, temperature, humidity, ROS elements, host type, and organic and inorganic pollution (Gadd et al., 2007; Frankland et al., 2009; Rodriguez-Romero et al., 2010). Several studies have reported on the impacts of climate change on fungus–host interactions, fungal distribution, fungal disease prevalence, and the production of fungal products (Frankland et al., 2009; Merrild, 2013; Botana and Sainz, 2015; Tiquia-Arashiro and Grube, 2019; Charters, 2020; Meyer et al., 2020; Frías-De-León et al., 2022; Miranda-Apodaca et al., 2023). The wide distribution and ecological diversity of Sordariomycetes fungi contributes to their frequent appearances in reports on the impact of climate change on fungal diversity, which occur especially frequently in climate-sensitive regions or populations of organisms that are closely associated with fungi.

Fungal pathogens that infect economically important crops, such as species of *Fusarium* (Vaughan et al., 2016) and *Magnaporthe* (Qiu et al., 2022), are of special concern. Epidemics of fungal pathogens can be enhanced by particularly wet and warm environments occurring during the flowering seasons of some crops. Remote sensing/geographic information system (GIS) technology has the potential to monitor snowline elevation, average temperature, precipitation, and sunshine hours in terms of their relationship with the product of *Cordyceps sinensis* (syn. *Ophiocordyceps sinensis*), which is also known as “yartsa gunbu” or “Dong Chong Xia Cao” (“winter worm summer grass”) in traditional Tibetan and Chinese medicine (Zhu et al., 2017). A comprehensive collection dataset and an ensemble species distribution modeling method have recently been used to investigate whether and how climate change affects the distribution of *C. sinensis*, and to predict potential shifts in the range of the fungus in the medium term of approximately 50–70 years in response to climate change (Yan et al., 2017; Li et al., 2019b). It has also been proposed that *Trichoderma* spp. could be utilized as “plant savers” in the face of climate change, on the basis of their several traits and genes that confer beneficial effects on crop plants (Kashyap et al., 2017). A limitation of these studies—with regard to their potential as a basis for omics-based analyses—is that they have thus far mainly addressed the ecological and diversity aspects of the global ecosystem. Hence, substantial fungal genomic resources that may illuminate potential responses to climate change have yet to be identified.

This would be a timely moment to revisit the possibility of developing *N. discreta*, along with other Sordariomycetes, as a model species to test the idea that adaptation to warm temperatures could lead to more efficient carbon metabolism (Romero-Olivares et al., 2015). To best adapt to their environments (often microenvironments), fungi have evolved elegant mechanisms for sensing and quickly responding to changes, and some of their genetic elements may be capable of high rates of evolution (e.g., Wang et al., 2016c; Chandra Nayaka et al., 2021; Kusch et al., 2023). Given the ecological and phenotypic diversity among closely related lineages within Sordariomycetes, further study and comparison of their potential for rapid genomic evolution would clarify their potential roles as models to enhance our understanding of long- and short-term climate and environmental impacts on fungi, and associated plant and animal health. As a motivating example, the relative abundance of *Fusarium* spp. has risen almost fivefold alongside the course of global warming (Delgado-Baquerizo et al., 2020).

We have only reviewed a very small portion of the recent comparative genomics and transcriptomics literature focusing on members of the class of Sordariomycetes, but these clearly illustrate the potential scientific rewards of comparing multiple genomes in different experimental settings to understand the evolution of the relevant organisms or traits and the genetics of select biological or ecological phenomena. However, the insights obtained on the basis of genomics/transcriptomics into trait evolution and into the associations with the genetics of various traits are still far from complete. Two possible factors that may significantly contribute to this gap are our underdeveloped understanding of the evolution of gene expression, and our lack of a system for integrating data from trait evolution and genetics that have already been (and can now be) intensively studied. Therefore, a large system encompassing well-studied model species, such as the Sordariomycetes, is a promising platform for the development of tools and the implementation of strategies to develop a systematic genetic understanding of trait evolution.

## Challenges and prospects for Sordariomycetes model systems in the omics era

Members of Sordariomycetes are model species and serve as key references for the annotation of newly sequenced non-model genomes. Nevertheless, investigation into the genomics and transcriptomics of the Sordariomycetes continues to reveal deeper layers of mysteries, including yet-to-be-resolved complexity, variation, and surprises. These efforts often encounter difficulties owing to the inherent limitations of currently available genomics/transcriptomics and bioinformatics technologies. Such limitations can be overcome, in part, by community efforts to invest in improving the annotation of the model genomes. Phylostratigraphy is a methodology that provides statistical descriptions of the origins of genes in a genome through homology-based searching across the species phylogeny (Domazet-Loso et al., 2007) with much more inclusive phylogenetic representation of genomes; the use of this approach has significantly reduced the number of *N. crassa* lineage-specific genes identified, and has demonstrated that they are clustered and aggregated toward the telomeres of each chromosome (Wang et al., 2022e; Wang et al., 2023). A recent examination of transcription factors in fungal genomes using homology searches identified a large number of mis-annotations as a result of sequencing errors and the mis-assembly of reference genomes (Mayer et al., 2023). However, historical genomic mis-annotations have been resolved only in the case of a few genomes, mainly because of the transient nature of the financial support for scientific advancements and accompanying technical difficulties. Usually, comparative genomic analyses focus on annotated gene families, functional groups, and specific questions about those genomes; thus, large amounts of the available data have been overlooked and are not likely to be revisited if this type of approach to the research persists. Although we anticipate the continued provision of resources for the analysis of larger numbers of new genomes, there is a greater need for support for the generation and comparative analysis of genomes of closely related organisms, so as to empower the development of model clades in terms of both a higher density of sampling and the generation of higher-quality comparisons and annotations. Clusters of classic models with a set of high-quality “core genomes” will be critical for a broader and more profound understanding of evolution of fungal and organismal diversity.

In addition to high-quality genomics data, there is a need for a solid basis for the design and development of methods for the analysis of high-quality transcriptomics, proteomics, and metabolics data from an evolutionary perspective (Jaffe et al., 2004; Nesvizhskii, 2014; Naranjo-Ortiz and Gabaldón, 2020; Sarsaiya et al., 2021). For example, proteogenomics—which combines proteomics, genomics, and transcriptomics—has considerably improved the annotation of *S. macrospora* through the identification of over 104 “hidden” proteins and the correction of annotations for over 500 genes (Blank-Landeshammer et al., 2019). A proteogenomic approach was also used to identify the function of transporter protein in *N. crassa* (Rupa et al., 2018). In a recent study focusing on eukaryotic chromatin evolution, *N. crassa* was sampled, along with two yeast models and other eukaryotes, for phylogenetic and proteomic reconstruction (Grau-Bové et al., 2022). In addition to being a model of clock- and/or light-responsive metabolic regulation, *N. crassa* has also served as a platform for the development of genome-scale metabolic models (Chen and Loros, 2009; Dreyfuss et al., 2013; Hurley et al., 2014; Bayram et al., 2019). The Sordariomycetes genomes represent excellent resources for investigation of the dramatic diversity of ecology and biology within the class. Recently, many studies have generated pangenomics and pantranscriptomics data, including on multiple strains within species, and developed core elements of fungal genomes and transcriptomes (Plissonneau et al., 2018; Badet et al., 2020; Bao et al., 2022; Hyun et al., 2022; Vaughn et al., 2022). However, the power of omics approaches is limited by a lack of knowledge of baseline gene activities and their functional dynamics in natural settings—even in the case of intensively studied biological processes of well-annotated model species. Therefore, one challenge that requires future effort is the integration of data and knowledge among “closely related” species—noting that fungi referred to as closely related are often divergent from a most recent common ancestor dating back to nearly the same period as the most recent common ancestor of mammals (**Figure 2**).

**Figure 3:**
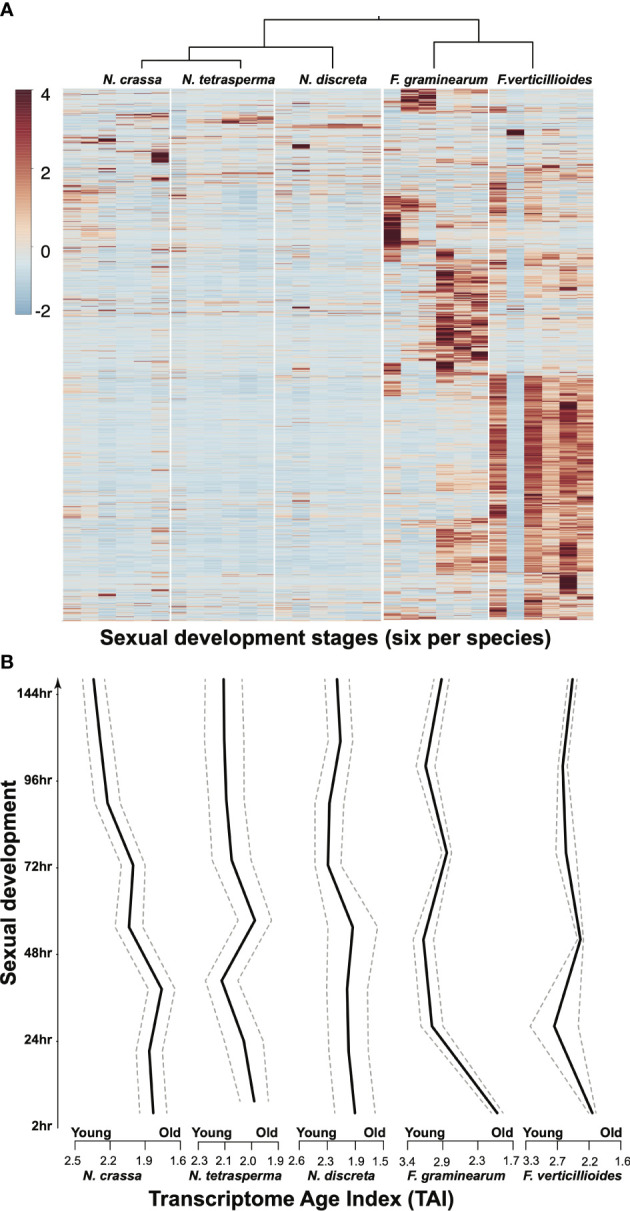
Comparative gene expression, illustrated in the form of **(A)** a heatmap and **(B)** a Transcriptome Age Index (TAI) plot (which combines gene phylogenetic age with expression changes to describe possible evo-developmental transcriptomics within a single genotype) for single-copy orthologous genes among five species during 144 h of sexual development. Gene expression data from our previous publications (Sikhakolli et al., 2012; Lehr et al., 2014; Trail et al., 2017) were revisited. TAI (the weighted mean of phylostrata using gene expression intensities of a given gene) was computed for each developmental (ontogenetic) stage following the methods described in previous studies on plant and animal models (Domazet-Lošo and Tautz, 2010; Drost et al., 2015).

Even under standard laboratory conditions, genome-level comparisons across closely related species face the need to deal with many unexpected results—even setting aside data heterogeneity resulting from the use of different experimental platforms by different research groups. In studies revisiting the transcriptomics data collected from three *Neurospora* and two *Fusarium* species over the morphologically conserved sexual reproduction process, large portions of the genomes have been found to exhibit divergent dynamics in terms of gene expression profiles (**Figure 3**). Such divergent activities have also been observed among different age groups of genes, suggesting that much more evolution of gene roles occurs than the conservation of functional groups implied by the current use of tools for functional annotation based on gene families and orthologous groups. One may argue that mRNA abundance may not directly reflect the functional status of the coding gene, meaning that additional well-sampled proteomics data are required to piece the puzzle together. This additional research is surely warranted: there is a lack of models with well-sampled transcriptomics and proteomics data covering key growth and developmental stages under standard laboratory conditions as well as more realistic conditions that better match natural environments. The establishment of a diverse range of comparative omics studies of the Sordariomycetes could enable intriguing and novel cross-class comparisons to be made. In addition, insights obtained from such analyses could well serve as starting points for novel hypothesis-driven experiments that can be carried out with relative ease in historically and newly amenable model species.

A central problem in genetics research is that of deciphering how genomic variation affects the function of genes and results in altered phenotypes. Results from genome-wide association studies (GWASs) with fungal genome data offer insights into the genetic basis of phenotypes or traits of interest. However, increasingly innovative methods will need to be developed to effectively integrate diverse data types and/or sources of information in order to identify functional genes and variants and understand how they shape the relevant phenotypes. These methods will probably include approaches that enable a move from detection of a genetic association signal in a chromosomal region to the identification of trait-associated genes and causal variants, as a step toward understanding the underlying morphological, ecological, or metabolic processes in fungi. Fine mapping, sequencing, functional studies, and other approaches have also been used to find the causal variants involved in complex traits, facilitated by statistical approaches to the integration of diverse data sources, including transcriptomics data from different cohorts, to interrogate causal relationships between genetic variation and the associated mechanism in fungal biology. The use of systems and synthetic biology approaches would also be a useful and creative way to investigate large-scale genetic and environmental interactions (G×E) within the genomes, especially among different genera that flourish in different environmental conditions. Additional obstacles to modeling in the class include: (1) the imbalanced sampling of genomes within this large and diverse fungal class, given that most of the available genomes are drawn from only four out of 45 orders, namely, Xylariale, Hypocreales, Sordariales, and Glomerellales; (2) the fact that many Sordariomycetes species are symbiotic and have not yet been successfully cultured under laboratory conditions; and (3) a lack of understanding of the ecology and biology of the model species in their natural settings. These challenges may partly be addressed by promoting collaboration among the relevant experts within and beyond our immediate scientific community and by incorporating developments in artificial intelligence (AI) that promise to accelerate genomics research.

On the latter point, the first critical step is the systematic feeding of diverse and high-quality data into AI tools, enabling computational facilitation based on reference data and data mining for applications ranging from genome annotation to systems biology analysis. Researchers have developed many widely used high-throughput computational methods of data analysis, including single-cell sequencing, to provide start-to-finish analytical ecosystems for large-scale omics datasets. These data are generated from experiments involving a wide range of systems that are considered to be of great interest for society (Hotaling et al., 2023). Models using methods such as manifold learning and deep learning have been developed, employing supervised and unsupervised learning approaches to the processing and visualization of data, the development of the field’s understanding of biological processes, and the characterization of phenotypic diversity and its underlying causal mechanisms. With their high-quality model genomes accompanied by high-quality omics data, the Sordariomycetes can be established at the core of omics databases, serving as a powerful set of model species for AI training for the analysis of evolutionary genomics, as has been pioneered in certain other disciplines (Guhlin et al., 2017; Misra et al., 2019; Biswas and Chakrabarti, 2020; Ko et al., 2020; Reel et al., 2021). Nevertheless, it is important to note that just as some erroneous gene annotations have been made and perpetuated over time, the quality of AI output, especially the outputs of new AI tools, which will be trained for new data types, will have to be critically monitored to minimize the embedding of historical misinterpretations and misconceptions that could substantially impede downstream science.

Big data provides us with the opportunity to address existing and new challenges in evolutionary genomics research. As omics data accumulate, the systematic investigation of genome–phenome relationships has expanded from model-driven gene-by-gene studies to data-driven studies of multiple species and multiple functional groups. This review has provided only a smattering of examples that touch on the substantial successes to be achieved by revealing the rules that underlie the relationships between genomes and phenomes. We advocate for the adoption of the class Sordariomycetes as a cluster of high-quality and diverse models that will enable extensive scientific advances capitalizing on Big Data in evolutionary genomics and that can be used as a high-level reference and blueprint for the analysis of other classes in the fungal kingdom and beyond. The richness and diversity of its members; their ecological capabilities, lifestyles, and economic importance; the accumulated research history with amenable model species; the abundance of sequenced genomes; the rapidly accumulating diversity of other omics data; and, last but not least, the immense global research community in this domain promise a synergy that will yield extraordinary and exciting scientific outcomes.

**Box 1: Glossary of technical terms**

Model organism: a species whose biology has been widely studied at many different levels and from different perspectives, serving as reference or basis of comparison for many other species. A model species often has particular experimental advantages, is usually easy to maintain, has a short generation time, and can breed and be manipulated in a laboratory setting.

Evolutionary genomics: the study of how features or components of a genome change both within and between species over evolutionary timescales, especially along lineages showing the divergence or convergence of interesting phenomes.

Comparative genomics: the direct comparison of the complete genetic material of one organism with that of another, or those of many others, to gain a better understanding of how genomes and species have evolved and to determine the functions of genes, transposable elements, and non-coding regions of genomes.

Omics: a set of methodologies targeting the collective qualification and quantification of pools of biological molecules, including genomic DNA (genomics), RNA (transcriptomics), proteins (proteomics), and metabolites (metabolomics), which translate into the structure, function, and regulation of an organism at different levels of dynamics.
