# Corrigendum: The Sordariomycetes: an expanding resource with Big Data for mining in evolutionary genomics and transcriptomics

**DOI:** 10.3389/ffunb.2024.1478516

**Published:** 2024-08-20

**Authors:** Zheng Wang, Wonyong Kim, Yen-Wen Wang, Elizabeta Yakubovich, Caihong Dong, Frances Trail, Jeffrey P. Townsend, Oded Yarden

**Affiliations:** ^1^ Department of Biostatistics, Yale School of Public Health, New Haven, CT, United States; ^2^ Korean Lichen Research Institute, Sunchon National University, Suncheon, Republic of Korea; ^3^ Department of Plant Pathology and Microbiology, The Robert H. Smith Faculty of Agriculture, Food and Environment, The Hebrew University of Jerusalem, Rehovot, Israel; ^4^ Institute of Microbiology, Chinese Academy of Sciences, Beijing, China; ^5^ Department of Plant Biology, Michigan State University, East Lansing, MI, United States; ^6^ Department of Plant, Soil and Microbial Sciences, Michigan State University, East Lansing, MI, United States; ^7^ Department of Ecology and Evolutionary Biology, Program in Microbiology, and Program in Computational Biology and Bioinformatics, Yale University, New Haven, CT, United States

**Keywords:** Sordariomycetes, evolution, genomics, transcriptomics, Big Data, *Neurospora*, *Fusarium*, *Trichoderma*

In the published article, there was an error in the **Funding** statement. We omitted mention of one of the grants supporting this study, IOS 1916137. The **Funding** originally read:

“The work of ZW, Y-WW, and JT was supported by funding awarded to JT by the National Institutes of Health R01, grant AI146584, and by the National Science Foundation, grant IOS1457044; the work of EY and OY was supported by funding award BSF-2018712 to OY. The work of CD was supported by the National Natural Science Foundation of China (32272786 and 31872163). FT was supported by the National Institutes of Health, R01 grant AI146584, and the Michigan State University AgBioResearch. The work of WK and FT was supported by the National Science Foundation, IOS 1456482, awarded to FT. WK was partly supported by the Basic Science Research Program through the National Research Foundation of Korea, funded by the Ministry of Education (2019R1I1A1A01057502).”

The correct **Funding** statement appears below.

“The work of ZW, Y-WW, and JT was supported by funding awarded to JT by the National Institutes of Health R01, grant AI146584, and by the National Science Foundation, grants IOS 1457044 and IOS 1916137; the work of EY and OY was supported by funding BSF-2018712 to OY. The work of CD was supported by the National Natural Science Foundation of China (32272786 and 31872163). FT was supported by the National Institutes of Health, R01 grant AI146584, and the Michigan State University AgBioResearch. The work of WK and FT was supported by the National Science Foundation, IOS 1456482, awarded to FT. WK was partly supported by the Basic Science Research Program through the National Research Foundation of Korea, funded by the Ministry of Education (2019R1I1A1A01057502).”

The authors apologize for this error and state that this does not change the scientific conclusions of the article in any way. The original article has been updated.

